# Cytomegalovirus Infection Is Associated with Development of Chronic Lung Allograft Dysfunction

**DOI:** 10.1007/s00408-022-00551-0

**Published:** 2022-07-06

**Authors:** David Bennett, Laura Bergantini, Pierluigi Ferrara, Maria Grazia Cusi, Sabino Scolletta, Francesca Montagnani, Piero Paladini, Piersante Sestini, Rosa Metella Refini, Luca Luzzi, Antonella Fossi, Elena Bargagli

**Affiliations:** 1 Respiratory Diseases Unit, University Hospital of Siena (AOUS), Viale Bracci, 16, 53100 Siena, Italy; 2 Department of Medical and Surgical Sciences & Neurosciences, University of Siena, Siena, Italy; 3 Virology Unit, University Hospital of Siena (AOUS), Siena, Italy; 4 Department of Medical Biotechnologies, University of Siena, Siena, Italy; 5 Emergency-Urgency and Organ Transplant, Anesthesia and Intensive Care, University Hospital of Siena (AOUS), Siena, Italy; 6 Infectious and Tropical Diseases Unit, University Hospital of Siena (AOUS), Siena, Italy; 7 Thoracic Surgery, University Hospital of Siena (AOUS), Siena, Italy; 8 Lung Transplant Unit, University Hospital of Siena (AOUS), Siena, Italy

**Keywords:** Cytomegalovirus, Chronic lung allograft dysfunction, Lung transplant, Clinical outcome

## Abstract

**Background:**

Cytomegalovirus (CMV) is the major and most common opportunistic infection complicating lung transplant (LTX). The aim of this study was to analyse the epidemiological aspects of CMV infection in lung transplant patients subject to a pre-emptive anti-CMV approach and to study the impact of this infection on lung transplant outcome, in terms of onset of chronic lung allograft dysfunction (CLAD).

**Methods:**

This single-centre retrospective study enrolled 87 LTX patients (median age 55.81 years; 41 females, 23 single LTX, 64 bilateral LTX). All patients were managed with a pre-emptive anti-CMV approach. The incidences of the first episode of CMV infection, 1, 3, 6 and 12 months after LTX, were 12.64%, 44.26%, 50.77% and 56.14%. A median interval of 41 days elapsed between LTX and the first episode of CMV infection. The median blood load of CMV-DNA at diagnosis was 20,385 cp/ml; in 67.64% of cases, it was also the peak value. Patients who had at least one episode had shorter CLAD-free survival. Patients who had three or more episodes of CMV infection had the worst outcome.

**Results:**

CMV infection was confirmed to be a common event in lung transplant patients, particularly in the first three months after transplant. It had a negative impact on transplant outcome, being a major risk factor for CLAD. The hypothesis that lower viral replication thresholds may increase the risk of CLAD is interesting and deserves further investigation.

## Introduction

Among the opportunistic infections that complicate lung transplant (LTX), cytomegalovirus (CMV) is the most common and important, being a major cause of morbidity and mortality [1–4]. In immunosuppressed patients, CMV infection can be asymptomatic but more often presents as CMV syndrome (fever, malaise, leukopenia, thrombocytopenia, high serum transaminase, etc.) or as specific CMV organ disease (pneumonia, gastroenteritis, hepatitis, etc.) [5]. Cytomegalovirus may also have various indirect effects on lung grafts and may be a risk factor for chronic lung allograft dysfunction (CLAD) [6, 7], the leading cause of mortality in LTX patients in the medium-to-long term [8, 9].

Two strategies are currently used to prevent CMV disease after solid organ transplant: antiviral prophylaxis and pre-emptive approach. These are not mutually exclusive; indeed, some transplant centres use both and the efficacy seems to be similar; however, international guidelines suggest antiviral prophylaxis for 6–12 months for patients undergoing lung transplant [9]. Antiviral prophylaxis involves administration of drugs with antiviral activity (ganciclovir, valganciclovir and possibly CMV-specific immunoglobulins) for a defined period to all patients at risk. Pre-emptive strategy, on the other hand, needs periodic monitoring of viral replication and administration of antiviral therapy only to patients with active CMV infection (i.e. with viral replication over a given threshold) to prevent infection from progressing to specific CMV-related organ disease [9].

The aim of this study was to analyse the epidemiological aspects of CMV infection in lung transplant patients subject to a pre-emptive anti-CMV approach and to study the impact of this infection on lung transplant outcome, in terms of onset of CLAD.

## Methods

### Population

In this single-centre retrospective study, we enrolled all patients who underwent LTX at the Lung Transplant Centre of Siena University Hospital between 2 January 2012 and 4 May 2021. A pre-emptive preventive strategy against CMV disease was adopted for all patients (diagnosis of CMV infection was considered when whole DNA-PCR greater than 10,000 cp/ml; see specific section). Patients who survived less than 30 days after LTX and those with donor-positive/recipient-negative CMV serology who received prophylactic antiviral therapy were excluded from the study. We collected all CMV-DNA PCR determinations carried out in our virology laboratory in the same period (*n* = 3106).

Patients were divided between those who had no episodes of CMV infection (Group 1) and those who had at least one episode (Group 2). Diagnosis of CMV infection was based on significant viral replication in blood, defined by quantitative DNA PCR greater than 10,000 cp/ml. The patients in Group 2 were in turn divided into three subgroups defined as follows: (2a) patients who had only one episode, (2b) patients who had only two episodes, and (2c) patients who had three or more episodes of post-transplant CMV infection.

The chronological series of CMV-DNA determinations in blood was analysed for each patient. It was decided that an episode of CMV infection began when CMV-DNA load was equal to or greater than 10,000 cp/ml and ended when it became equal to or less than 500 cp/ml.

Of the 101 patients who underwent LTX at our centre in the study period, 87 were included in the study (during study period 9 patients died within 30 days from transplantation and 5 were excluded because of D+/R− serology). The median age of patients was 55.81 years (lower 95% CI 47.58; upper 95% CI 53.31 years), 46 were male and 41 female. All patients gave their informed consent to participation in the study (Study Respir1, Prot n 15732).

The following preoperative data were collected from all patients: age at LTX, gender, underlying lung disease, body mass index (BMI), history of smoking, comorbidities, time on LTX waiting list and extracorporeal membrane oxygenation (ECMO) bridge-to-LTX, as well as the following intraoperative data: single/bilateral LTX, blood transfusions, cardiovascular failure, recourse to ECMO (in all cases of poor haemodynamic control and low intraoperative oxygenation, veno-arterial ECMO with central cannulation was used), induction therapy, prolonged invasive mechanical ventilation (IMV) (> 96 h), tracheostomy, primary graft dysfunction grade 72 h after transplant, recourse to postoperative ECMO, time on vasoactive amine therapy, time on nitric oxide inhalation therapy, time in intensive care and total hospital stay.

### Immunosuppression Protocol

Induction therapy was administrated in 65/87 patients: 62 patients were given basiliximab and three thymoglobulin. The decision on whether to administer induction therapy and which drug to use was left to the surgeon in charge. All patients received immunosuppressant therapy with corticosteroids, a calcineurin inhibitor (tacrolimus) and mycophenolate mofetil.

### Anti-CMV Protocol

A pre-emptive approach to the prevention of CMV disease after LTX was used in all cases. In recipient-negative/donor-positive cases, antiviral prophylaxis was performed with intravenous ganciclovir, followed by oral valganciclovir for at least 6 months after transplant, and subsequently according to the conventional pre-emptive approach. Patients who were recipient negative/donor positive (R−/D +) were excluded from the present study.

The pre-emptive approach envisages laboratory surveillance with periodic determination of blood concentrations of CMV-DNA. The latter are determined by quantitative PCR on whole blood rendered uncoagulable by adding calcium citrate to the sample. In the first month after LTX, CMV-DNA was determined every 3–4 days, in the next 2 months every 7–10 days and subsequently monthly for the first year and thereafter every 2–3 months and based on clinical conditions. In our centre, CMV-DNA PCR load at 10,000 cp/ml or above is considered predictive of CMV disease and antiviral therapy is routinely given.

### Statistical Analysis

Statistical analysis was conducted with StatSoft (2001) (TIBCO Software, Palo Alto, CA, USA) and GraphPad Prism v 9.0.2 software (GraphPad Software, San Diego, CA, USA). Comparisons of continuous variables were performed with non-parametric tests and the differences with *p* ≤ 0.05 were considered significant. Specifically, comparisons between two groups were performed with the Mann-Whitney U test, while comparisons between more than two groups were performed by Kruskal-Wallis test. Contingency table comparisons were performed with the Chi-square test or Fisher’s exact test. All data were expressed as median (lower 95% CI upper 95% CI), unless otherwise indicated. Survival analysis was performed using Kaplan–Meier curves.

## Results

The present study included 87 of the 101 patients who underwent LTX at our centre in the period 2 January 2012–4 May 2021 [median age = 55.81 years (lower 95% CI 47.58; upper 95% CI 53.31 years); 46 males, 41 females (23 single LTX and 64 bilateral LTX)].

The determinations of blood CMV-DNA by PCR numbered 3106: 34 determinations per patient (median) (lower 95% CI 31.65; upper 95% CI 39.75; minimum = 2; maximum = 94).

Of the 87 patients included in the study, 68 episodes of CMV infection were identified. In 43 cases, it was the first post-transplant infection, 18 cases were the second episode of infection, six cases were the third episode and one case was the fourth episode. Thus, 43/87 (49.43%) patients had at least one episode of CMV infection: 25/87 (28.74%) had only one episode, 12/87 (13.79%) had two episodes and 6/87 (6.90%) had three or more episodes.

The median viral load of CMV-DNA at the beginning of the episode of CMV infection (i.e. at the time of diagnosis) was 20,385 cp/ml (lower 95% CI 22,979; upper 95% CI 34,742); in 46/68 (67.64%) episodes of CMV infection, the value of the first determination was also the peak value.

A median time of 41 days (lower 95% CI 40.72; upper 95% CI 104.7) elapsed between LTX and the first episode of CMV infection. Of the 43 first episodes of CMV infection, 11 occurred within 1 month of LTX (25.58%), 26 occurred between the 2nd and 3rd months (60.46%), three occurred between the 4th and 6th months (6.97%), one case occurred between the 7th and 12th months (2.34%) and two cases occurred in more than 1 year after transplant (4.65%). The incidences of the first episode of CMV infection at 1, 3, 6 and 12 months after LTX were 12.64%, 44.26%, 50.77% and 56.14%, respectively.

We observed at least one second episode of CMV infection in 25 patients. Overall, of the 68 episodes of CMV infection, 56 (82.35%) resolved completely in a median time of 31.5 days [lower 95% CI 29.57; upper 95% CI 62.14], whereas nine (13.24%) did not resolve because the patient died during the CMV infection. Of these, seven died with a viral load of CMV-DNA > 10,000 cp/ml and two died with CMV-DNA between 500 and 5000 cp/ml. Three (4.41%) patients were of unknown outcome because the study time frame stopped during the infection; 8.05% of patients died with ongoing CMV infection.

Comparisons of preoperative, intraoperative and postoperative variables of the study groups are shown in Tables 1, 2, 3 and 4.

**Table 1:** Preoperative data

		Group 1	Group 2	*p*-value
Patients	*n*	44	43	
Age (years) at LTX	median	48.71	57.21	0.0220*
	(L95%CI–U95%CI)	(42.70–51.71)	(50.36–57.18)	
Males	*n*	18	28	0.0320*
	(%)	(40.91)	(65.12)	
Diagnosis indicating LTX				
Pulmonary fibrosis	*n*	16	23	0.2045
	(%)	(36.36)	(53.49)	
COPD	*n*	7	8	
	(%)	(15.91)	(16.60)	
Cystic fibrosis	*n*	14	6	
	(%)	(31.82)	(13.95)	
Other	*n*	7	6	
	(%)	(15.91)	(13.95)	
BMI (kg/m^2^)	Median	22.75	23.00	0.3635
	(L95%CI–U95%CI)	(21.54–24.60)	(22.47–25.66)	
History of smoking	*n*	15	21	0.1948
	(%)	(34.09)	(48.84)	
Comorbidities				
Diabetes mellitus	*n*	16	14	0.8223
	(%)	(36.36)	(32.56)	
Arterial hypertension	*n*	14	14	0.9999
	(%)	(31.82)	(32.56)	
Hypercholesterolemia	*n*	11	16	0.2523
	(%)	(25.00)	(37.21)	
Osteoporosis	*n*	30	25	0.3786
	(%)	(68.18)	(58.14)	
Time (days) on LTX waiting list	median	260.5	201.0	0.1673
	(L95%CI–U95%CI)	(270.3–512.9)	(197.1–376.8)	
ECMO-bridge-to-LTX	*n*	7	4	0.5210
	(%)	(15.91)	(9.30)	

Patients were divided between those who had no episodes of CMV infection (Group 1) and those who had at least one episode (Group 2)

*LTX* lung transplant, *COPD* chronic obstructive pulmonary disease, *BMI* body mass index, *ECMO* extracorporeal membrane oxygenation

*Statistically significant

**Table 2:** Intra- and postoperative data

		Group 1	Group 2	*p*-value
Patients	*N*	44	43	
Type of LTX				
Single	*N*	8	15	0.0927
	(%)	(18.18)	(34.88)	
Bilateral	*n*	36	28	
	(%)	(81.82)	(65.12)	
Induced immunosuppression	*n*	34	31	0.6281
	(%)	(77.27)	(72.09)	
Cardio-circulatory shock	*n*	5	1	0.2024
	(%)	(11.36)	(2.33)	
Time (hours) on vasoactive amine therapy	Median	64.00	72.00	0.2363
	(L95%CI–U95%CI)	(50.06–112.4)	(41.39–234.6)	
Blood transfusions	*n*	21	18	0.6681
	(%)	(47.73)	(41.86)	
IMV > 96 ore	*n*	14	17	0.5065
	(%)	(31.82)	(39.53)	
Tracheostomy	*n*	10	7	0.5902
	(%)	(22.73)	(16.28)	
Time (hours) on NO inhalation therapy	Median	48.00	48.00	0.4267
	(L95%CI–U95%CI)	(39.13–102.9)	(39.35–73.96)	
Intraoperative ECMO	*n*	11	12	0.8112
	(%)	(25.00)	(27.91)	
Postoperative ECMO	*n*	6	2	0.2656
	(%)	(13.64)	(4.65)	
PGD grade at 72 h				
Grade 0	*n*	10	5	0.1198
	(%)	(22.73)	(11.63)	
Grade 1	*n*	15	11	
	(%)	(34.09)	(25.58)	
Grade 2	*n*	12	11	
	(%)	(27.27)	(25.58)	
Grade 3	*n*	7	16	
	(%)	(15.91)	(37.21)	
Time (days) in intensive care	Median	9.00	9.00	0.5990
	(L95%CI–U95%CI)	(12.98–23.81)	(10.48–22.15)	
Time (days) in hospital	Median	36.00	37.00	0.4161
	(L95%CI–U95%CI)	(38.22–54.71)	(36.78–48.00)	

Patients were divided between those who had no episodes of CMV infection (Group 1) and those who had at least one episode (Group 2)

*LTX* lung transplant, *IMV* invasive mechanical ventilation, *NO* nitric oxide, *ECMO* extracorporeal membrane oxygenation, *PGD* primary graft dysfunction

**Table 3:** Preoperative data

		Group 1	Group 2			*p*-value
			Group 2a	Group 2b	Group 2c	
Patients	*N*	44	25	12	6	
Age (years) at LTX	Median	48.71	57.87	56.06	59.39	0.1010
	(L95%CI–U95%CI)	(42.70–51.71)	(49.01–58.58)	(44.06–58.90)	(50.80–65.63)	
Males	*n*	18	18	8	3	0.0685
	(%)	(40.91)	(72.00)	(66.67)	(50.00)	
Diagnosis indicating LTX						
Pulmonary fibrosis	*n*	16	10	8	5	0.2159
	(%)	(36.36)	(40.00)	(66.67)	(83.33)	
COPD	*n*	7	5	2	1	
	(%)	(15.91)	(20.00)	(16.67)	(16.67)	
Cystic fibrosis	*n*	14	4	2	0	
	(%)	(31.82)	(16.00)	(16.67)	(0.00)	
Other	*n*	7	6	0	0	
	(%)	(15.91)	(24.00)	(0.00)	(0.00)	
BMI (kg/m^2^)	Median	22.75	22.50	24.70	23.00	0.3312
	(L95%CI–U95%CI)	(21.54–24.60)	(20.98–24.95)	(22.34–29.15)	(17.10–31.65)	
History of smoking	*n*	15	12	7	2	0.3920
	(%)	(34.09)	(48.00)	(58.33)	(33.33)	
Comorbidities						
Diabetes mellitus	*n*	16	9	5	0	0.3174
	(%)	(36.36)	(36.00)	(41.67)	(0.00)	
Arterial hypertension	*n*	14	10	4	0	0.3134
	(%)	(31.82)	(40.00)	(33.33)	(0.00)	
Hypercholesterolemia	*n*	11	9	6	1	0.3040
	(%)	(25.00)	(36.00)	(50.00)	(16.67)	
Osteoporosis	*n*	30	13	8	4	0.5910
	(%)	(68.18)	(52.00)	(66.67)	(66.67)	
Time (days) on LTX waiting list	Median	260.5	199.0	225.5	204.0	0.5624
	(L95%CI–U95%CI)	(270.3–512.9)	(159.3–421.7)	(108.8–502.0)	(97.64–372.7)	
ECMO-bridge-to-LTX	*n*	7	2	2	0	0.5814
	(%)	(15.91)	(8.00)	(16.67)	(0.00)	

Patients who had no episodes of CMV infection (Group 1), patients who had at least one episode (Group 2). Group 2: (2a) patients who had only one episode of post-transplant CMV infection; (2b) patients who had only two episodes of CMV infection; (2c) patients who had three or more episodes of CMV infection

*LTX* lung transplant, *COPD* chronic obstructive pulmonary disease, *BMI* body mass index, *ECMO* extracorporeal membrane oxygenation

**Table 4:** Intra- and postoperative data

		Group 1	Group 2			*p*-value
			Group 2a	Group 2b	Group 2c	
Patients	*n*	44	25	12	6	
Type of LTX						
Single	*n*	8	9	3	3	0.2174
	(%)	(18.18)	(36.00)	(25.00)	(50.00)	
Bilateral	*n*	36	16	9	3	
	(%)	(81.82)	(64.00)	(75.00)	(50.00)	
Induced immunosuppression	*n*	34	16	11	4	0.2954
	(%)	(77.27)	(64.00)	(91.67)	(66.67)	
Circulatory failure	*n*	5	0	1	0	0.2955
	(%)	(11.36)	(0.00)	(8.33)	(0.00)	
Time (hours) on vasoactive amine therapy	Median	64.00	72.00	96.00	84.00	0.5008
	(L95%CI–U95%CI)	(50.06–112.4)	(− 16.68–265.1)	(− 28.68–390.9)	(− 17.47–173.5)	
Blood transfusions	*n*	21	9	7	1	0.2934
	(%)	(47.73)	(36.00)	(58.33)	(16.67)	
IMV > 96 h	*n*	14	7	8	2	0.1374
	(%)	(31.82)	(28.00)	(66.67)	(33.33)	
Tracheostomy	*n*	10	4	2	1	0.9014
	(%)	(22.73)	(16.00)	(16.67)	(16.67)	
Time (hours) on NO inhalation therapy	Median	48.00	48.00	72.00	24.00	0.6289
	(L95%CI–U95%CI)	(39.13–102.9)	(34.55–85.56)	(30.11–98.62)	(11.45–30.55)	
Intraoperative ECMO	*n*	11	5	6	1	0.2309
	(%)	(25.00)	(20.00)	(50.00)	(16.67)	
Postoperative ECMO	*n*	6	0	2	0	0.1732
	(%)	(13.64)	(0.00)	(16.67)	(0.00)	
PGD grade 72 h						
Grade 0	*n*	10	4	0	1	0.5200
	(%)	(22.73)	(16.00)	(0.00)	(16.67)	
Grade 1	*n*	15	5	4	2	
	(%)	(34.09)	(20.00)	(33.33)	(33.33)	
Grade 2	*n*	12	7	3	1	
	(%)	(27.27)	(28.00)	(25.00)	(16.67)	
Grade 3	*n*	7	9	5	2	
	(%)	(15.91)	(36.00)	(41.67)	(33.33)	
Time (days) in intensive care	Median	9.00	8.00	11.00	7.00	0.5498
	(L95%CI–U95%CI)	(12.98–23.81)	(9.522–29.00)	(6.627–22.54)	(2.212–14.79)	
Time (days) in hospital	Median	36.00	36.00	48.00	38.00	0.8484
	(L95%CI–U95%CI)	(38.22–54.71)	(33.20–49.38)	(34.38–52.35)	(22.38–67.62)	

Patients who had no episodes of CMV infection (Group 1), patients who had at least one episode (Group 2). Group 2: (2a) patients who had only one episode of post-transplant CMV infection; (2b) patients who had only two episodes of CMV infection; (2c) patients who had three or more episodes of CMV infection

*LTX* lung transplant, *IMV* invasive mechanical ventilation, *NO* nitric oxide, *ECMO* extracorporeal membrane oxygenation, *PGD* primary graft dysfunction

Patients who had at least one episode of CMV infection (Group 2) were older (*p* = 0.0220) than patients who had no episodes (Group 1) and were predominantly males (*p* = 0.0320). No other significant differences in the variables considered emerged between the two groups or within the subgroups (2a, 2b and 2c).

**Fig. 1:**
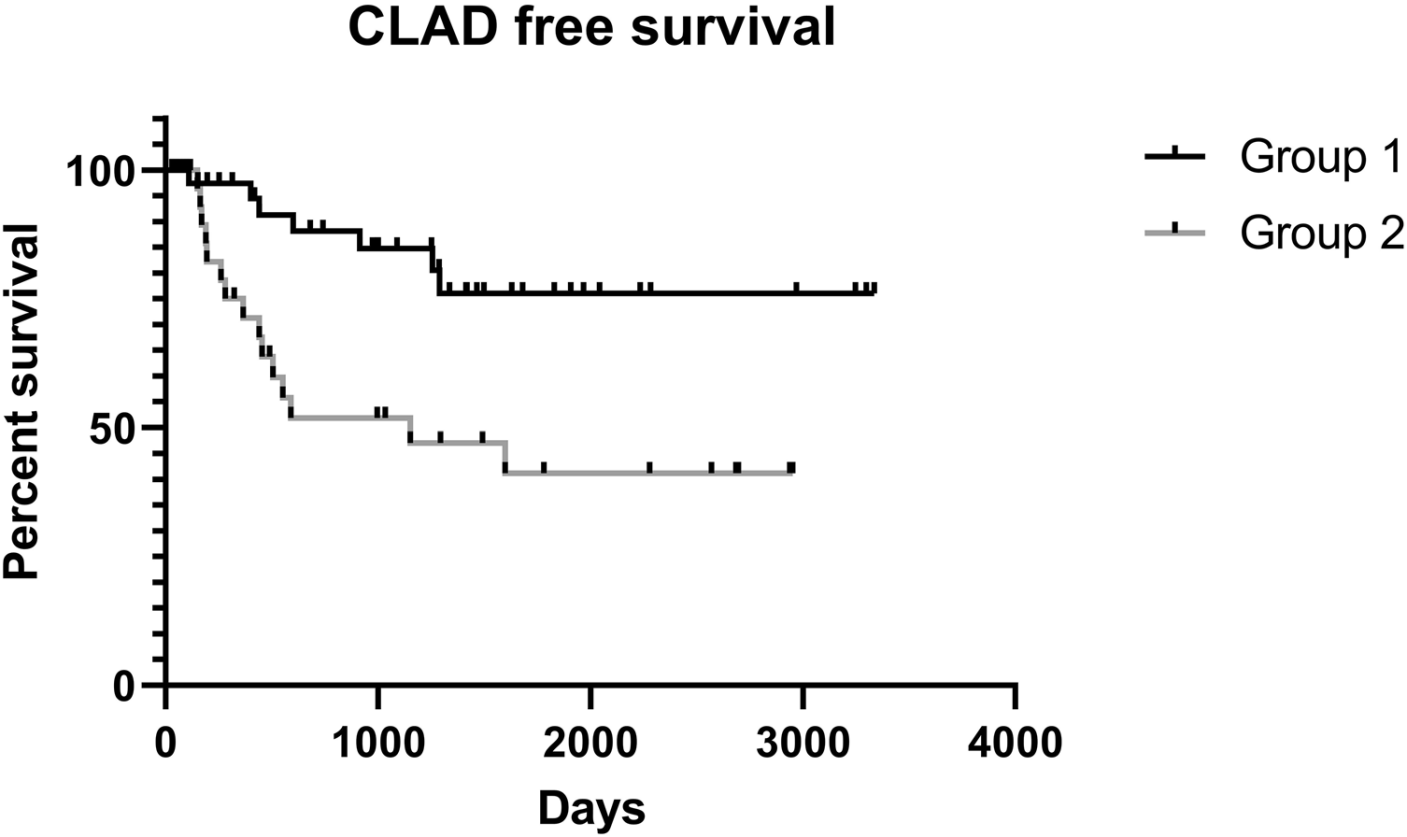
Kaplan–Meier CLAD-free survival curves of patients who had no episodes of CMV infection (Group 1) and patients who had at least one episode (Group 2) (*p* = 0.0001)

**Fig. 2:**
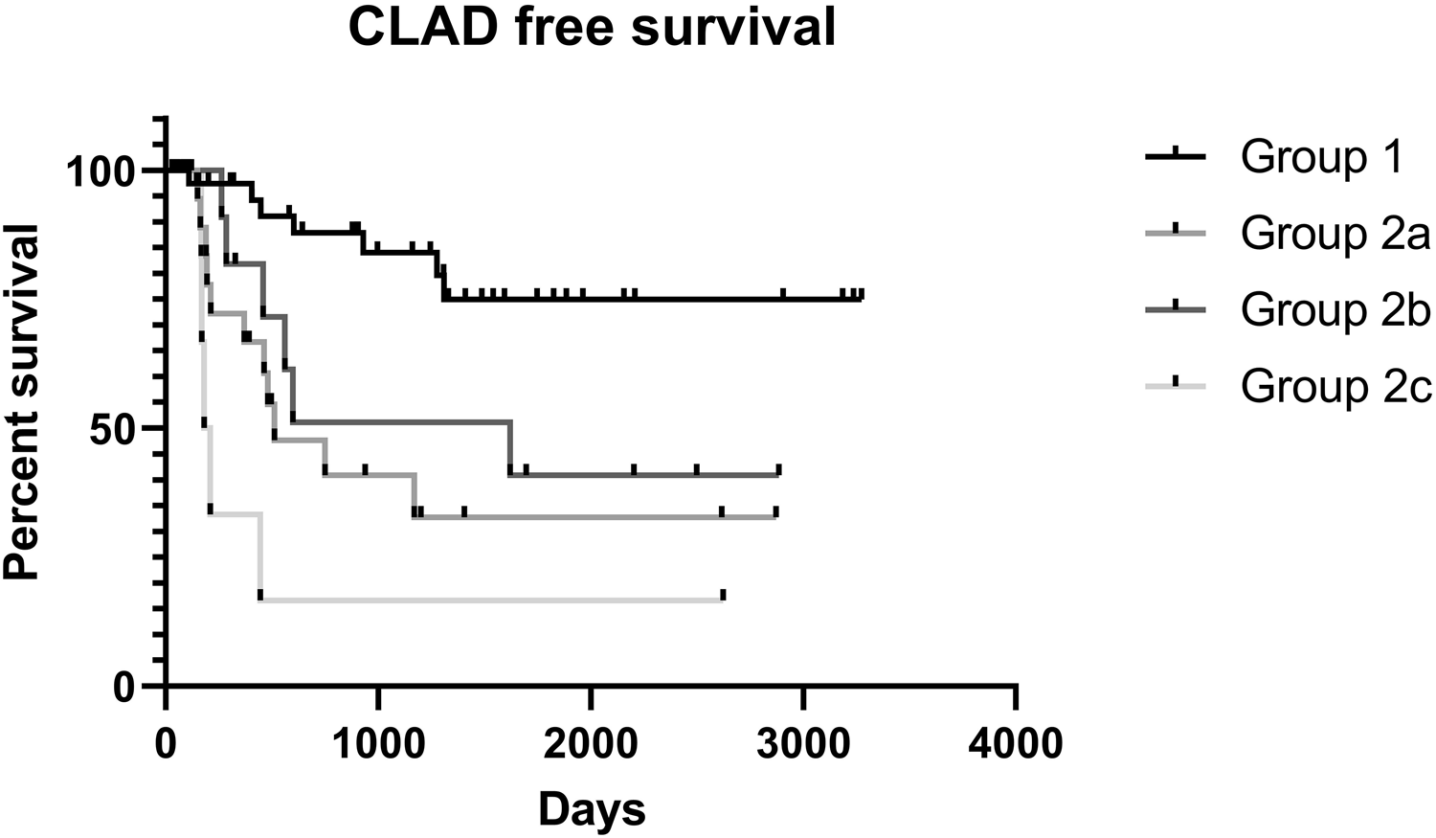
Kaplan–Meier CLAD-free survival curves of patients who had no episodes of CMV infection (Group 1), patients who had only one episode of post-transplant CMV infection (Group 2a), patients who had only two episodes of CMV infection (Group 2b) and patients who had three or more episodes of CMV infection (Group 2c) (*p* < 0.0001)

Regarding survival, we did not observe any statistically significant differences in overall survival curves between Groups 1 and 2 (*p* = 0.1848), or between subgroups (*p* = 0.2573). A statistically significant difference was observed in CLAD-free survival curves between Groups 1 and 2 (*p* = 0.0001; Fig. 1) (median Group 1: undefined, as percent of CLAD-free survival of the sample was greater than 50%; median Group 2: 1153 days), and among subgroups (*p* < 0.0001; Fig. 2). Specifically, compared with patients who had no episodes of CMV infection, patients who had at least one episode had shorter CLAD-free survival. Patients who had one (Group 2a) or two episodes of CMV infection (Group 2b) had similar CLAD-free survival curves, but shorter than those of patients who did not experience CMV infection (Group 1) and longer than those of patients who had three or more episodes of CMV infection (Group 2c), who had the worst outcome (medians undefined, 1153, 1598 and 305 days, respectively).

## Discussion

Cytomegalovirus infection is the major opportunistic infection complicating lung transplant [4, 6]. Among solid organ transplants, the highest incidence of CMV infection has been reported in patients undergoing lung transplant, at least partly due to the more aggressive immunosuppression induced [2, 9]. The aim of this study was to analyse the epidemiological aspects of CMV infection in patients undergoing lung transplant at the Lung Transplant Centre of Siena, Italy, and to explore its impact on the outcome of LTX.

We analysed episodes of CMV infection, diagnosed by determining viral loads of CMV-DNA by PCR in 87 lung transplant patients managed with a pre-emptive approach, observing an overall incidence of infection of 49.43%. This seems lower than the percentages reported in the literature, according to which the infection affects up to 75% of patients undergoing solid organ transplant [2]. It is difficult to say whether our data are underestimated because the particularities of viraemia determination methods make comparison with other centres problematical, moreover 5 out 101 initial patients were excluded from the study because D+/R−. Apart from the fact that some centres continue to use CMV antigenemia as a measure of viraemia, a technique that is certainly obsolete and less sensitive than gene amplification. The sensitivity of the various commercially available assays for the determination of blood concentrations of CMV-DNA can be different and the viraemia thresholds used by different centres to define an episode of CMV infection may also vary [2, 9, 10].

Most of the first episodes of CMV infection (86.05%) occurred within 3 months of transplant. This is in line with the literature and principally due to the high levels of immunosuppression to which patients are subject in the first months after transplant [2, 5]. Our data showed incidences of the first episode of CMV infection of 12.64% at 1 month, 44.26% at 3 months, 50.77% at 6 months and 56.14% at 12 months after LTX.

It is known that the main risk factors for CMV infection in solid organ transplant recipients concern donor/recipient serological matching, the organ transplanted (lung is the riskiest) and occurrence of acute rejection episodes [2, 9, 11]. However, in our series, we could only demonstrate the association with older age and male sex. It is difficult to interpret this finding, regarding age, it is reasonable to suppose that older patients, with less efficient immune systems, are more susceptible to immunosuppressive regimes.

Although we did not study the incidence of CMV-specific organ disease, but limited our consideration to analysis of laboratory values of PCR-CMV, it is interesting to note that the median viral load of CMV-DNA of our patients at the beginning of episodes of infection, i.e. at the time of diagnosis, was of the order of 10^4^ cp/ml (20,385; lower 95% CI 22,979; upper 95% CI 34,742) and that the first determination was also the peak value in most of cases (67.64%). Follow-up and treatment of CMV reactivation is not standardized, CMV PCR levels might fluctuate over time and not all Centres start treatment immediately, especially not if there are no symptoms. However, our pre-emptive protocol with periodic monitoring and prompt introduction of antiviral therapy, with quite low threshold also in absences of symptoms, showed to be effective in identifying patients with CMV infection and successful in quickly stopping viral replication.

Several studies have associated CMV infection with chronic rejection [6, 12–15]. Our study confirms these observations, finding a significant association between CMV infection and early development of chronic allograft dysfunction. The CLAD-free survival curves showed that (1) patients who had at least one episode of CMV infection had shorter CLAD-free survival curves; (2) patients who had one or two episodes of CMV infection had similar CLAD-free survival times, but shorter than those of patients who had no episodes and longer than those of patients who had three or more episodes of CMV infection, who showed the worst survival. The negative impact of CMV on the outcome of lung transplant has been attributed to the direct and indirect effects of the virus on the body and the graft [4, 5]. The direct effects are CMV disease, i.e. CMV syndrome and CMV end organ disease (possibly pneumonitis), whereas the indirect effects are due to the immunomodulatory properties of CMV, which increases the risk of developing other infections or of acute rejection and chronic lung allograft dysfunction [9, 11–15]. In this sense, active CMV infection is an independent predictor of mortality after solid organ transplant [9]. In immunosuppressed patients, CMV can reactivate from its sites of persistence and give rise to viraemia that can spread the infection to organs whose reduced local defences may lead to onset of disease. CMV viraemia is therefore a predictor of impending CMV disease in transplant patients [2, 5, 9]. Quantitative PCR has unequivocally demonstrated the existence of a sigmoidal relationship between the probability of CMV disease and viral load of CMV-DNA, a relationship which suggests that antiviral measures aimed at preventing CMV disease should be initiated for viral loads between 10^3^ and 10^4^ cp/ml [9].

There are numerous pros and cons for the two strategies of preventing CMV disease (i.e. antiviral prophylaxis versus preventive therapy based on a pre-emptive approach), of which cost, logistics and side effects of drugs have the greatest weight. The two strategies are not mutually exclusive: indeed, some transplant centres use both and the effectiveness seems to be similar in terms of reducing the risk of CMV disease [9]. Our data, however, suggest the need for greater protection of lung transplant patients to ensure that not even one episode of CMV infection occurs, as this is already a major risk factor for early onset of CLAD.

Considering the present results, our centre decided to review its anti-CMV prophylaxis policy, abandoning the pre-emptive approach and resorting to antiviral therapy with ganciclovir and subsequently valganciclovir in combination with human anti-CMV immunoglobulins. Indeed, in addition to therapy with antiviral chemotherapeutics, prophylaxis with human anti-CMV immunoglobulins has been proposed [15]. These measures suggest a role of humoral immunity, not only in the treatment of viral infection, but also in the reduction of acute rejection rates, probably through the potential indirect immunomodulatory activity of this compound [15–17].

The present study has some limitations, including the relatively small statistical sample and its retrospective nature, which did not allow us to evaluate the incidence of CMV-related organ disease. However, the most interesting finding is the clear association of CMV infection with earlier development of CLAD.

In conclusion, CMV infection is confirmed as a common event in lung transplant patients, particularly in the first 3 months after transplant. Our study confirms its negative impact on transplant outcome, being a major risk factor for CLAD. Prophylactic strategies capable of improving control of CMV are necessary to ensure better long-term success of lung transplants. Further studies on larger, multicentre and prospective case series are needed to better clarify the role of CMV infection in lung transplant outcome. The hypothesis that low viral replication thresholds, even lower than the cutoff used in this study, may have a decisive role in the development of CLAD is interesting and deserves further investigation for more timely and personalized therapeutic approaches.
